# Selenium Digestibility and Bioactivity in Dogs: What the Can Can, the Kibble Can’t

**DOI:** 10.1371/journal.pone.0152709

**Published:** 2016-04-04

**Authors:** Mariëlle van Zelst, Myriam Hesta, Kerry Gray, Karen Beech, An Cools, Lucille G. Alexander, Gijs Du Laing, Geert P. J. Janssens

**Affiliations:** 1 Department of Nutrition, Genetics and Ethology, Faculty of Veterinary Medicine, Ghent University, Merelbeke, Belgium; 2 WALTHAM^®^ Centre for Pet Nutrition, Waltham-on-the-Wolds, Leicestershire, United Kingdom; 3 Department of Applied Analytical & Physical Chemistry, Faculty of Bioscience Engineering, Ghent University, Ghent, Belgium; Wageningen UR Livestock Research, NETHERLANDS

## Abstract

There is a growing concern for the long-term health effects of selenium (Se) over- or underfeeding. The efficiency of utilization of dietary Se is subject to many factors. Our study in dogs evaluated the effect of diet type (canned versus kibble) and dietary protein concentration on Se digestibility and bioactivity. Canned and kibble diets are commonly used formats of dog food, widely ranging in protein concentration. Twenty-four Labrador retrievers were used and four canned and four kibble diets were selected with crude protein concentrations ranging from 10.1 to 27.5 g/MJ. Crude protein concentration had no influence on the digestibility of Se in either canned or kibble diets, but a lower Se digestibility was observed in canned compared to kibble diets. However, the biological activity of Se, as measured by whole blood glutathione peroxidase, was higher in dogs fed the canned diets than in dogs fed the kibble diets and decreased with increasing crude protein intake. These results indicate that selenium recommendations in dog foods need to take diet type into account.

## Introduction

Selenium (Se) is an essential trace element, primarily to protect against oxidative stress [1]. Current dietary recommendations include a safety margin to avoid loss of antioxidant protection at low concentrations and the risk of Se toxicity at high concentrations [2]. In addition, data on the importance of Se intake on long-term health are emerging that give rise to other concerns. There are indications that Se is involved in the prevention of diseases such as cancer [3–6] and cardiovascular diseases [7–9] and impaired immune function [10–11], etc. On the other hand, the Se and vitamin E cancer prevention trial (SELECT) reported an increased risk of diabetes mellitus type 2 in humans with a high Se intake (200 μg selenomethionine/day) [12]. The effects of selenium intake on the health and longevity of companion animals such as the dog is now also coming under scrutiny [13–14]. The current recommended allowance for Se in dog food takes into account a bioavailability factor, but is not diet type specific [15], despite several studies reporting a large variation in Se digestibility between diet types [16–19]. We have previously shown in 20 canned and 23 kibble diets, the average amount of Se was higher in canned than kibble diets (34.8 vs. 22.5 μg/MJ, respectively). Also, apparent crude protein (CP) digestibility had a significant effect on *in vitro* Se accessibility [19], i.e. the dietary Se fraction in the filtrate after *in vitro* digestion.

The reasons for the differences in Se digestibility between canned and kibble diets may be due to the way the diets are processed or the raw materials used during manufacture. The raw materials used for pet foods vary greatly in their Se concentration [20–21] and availability [22]. Canned diets typically contain higher concentrations of meat and/or meat by-products, whilst kibble diets contain mainly cereal grains and cereal grain by-products [21]. Wedekind *et al*. [17] tested the availability of Se in several pet food ingredients and found a range of 9% in mackerel to 38% in beef spleen when compared to the availability of sodium selenite. Due to the differences in ingredients, it is likely that the chemical form of Se in the diet will also be different between canned and kibble diets, which is known to be an important factor for the digestibility of Se [23–24].

Canned and kibble diets also differ in the way of processing. Kibble diets are extruded and canned diets are retorted, resulting in differential effects of heat, pressure and shear which may also impact on Se digestibility. The differences between canned and kibble diets may not only cause a variation in Se digestibility between diet types, but also the bioactivity, defined as the amount of Se that can be used for the incorporation into selenoproteins. The antioxidant glutathione peroxidase (GPx) is often used as a measure of Se bioactivity [25–29]. In addition, serum isoprostanes (IsoPs) are a measure for lipid peroxidation [30] and are used as an indicator of total antioxidant status. Selenium is not only incorporated into GPx, but also into iodothyronine deiodinases, of which type I and II are involved in the transformation of thyroxine (T4) into triiodothyronine (T3) [31–32]. Olivieri *et al*. [32] has shown that selenium status was positively correlated with the ratio of T3:T4 in humans and Wedekind *et al*. [28] have reported similar results in puppies.

Based on the *in vitro* results [19], it was hypothesized that canned diets have a lower Se digestibility than kibble diets and that CP concentration (as the main factor for the ingested amount of digestible protein) is positively associated with Se digestibility in kibble diets and negatively in canned diets *in vivo*. To test this hypothesis, two different diet types (canned and kibble) and four different concentrations of CP per diet type were selected. Diets were selected to reflect the processing conditions used in the pet food industry, including the characteristics that are associated with diet type and dietary CP concentration.

## Experimental Methods

### Study design

The study consisted of four feeding periods with six transition days and experimental periods of between 29–43 days. Four groups of each six dogs were selected. Each dog group was fed four of the eight experimental diets (2 canned, 2 kibble) in a randomised incomplete cross-over design. Blood, urine and faecal samples were taken at the end of every feeding period. This study (HO0647) was approved by the WALTHAM Centre for Pet Nutrition Animal Welfare and Ethical Review Body and was conducted under Home Office Project License authorisation.

### Dogs

Twenty-four adult Labrador retrievers, of which 15 female (14 neutered, 1 entire) and 9 male (all neutered), were selected for this study. All dogs used in this study were bred at WALTHAM or sourced from Home Office approved breeders for research purposes. At the start of the study, the average age of the dogs was 4.2 years (range 2.0–8.1) and the average body weight (BW) was 27.9 kg (range 23.7–32.7 kg). The dogs were fed twice daily (8:30 and 15:00) to maintain BW and body condition score (BCS) at ideal (D) on the S.H.A.P.E^™^ (Size, Health And Physical Evaluation) BCS-scale [33]. BW and BCS were recorded weekly and food intake daily. Dogs had access to fresh drinking water at all times. Dogs were divided into four treatment groups of six dogs, with age, BW and gender as blocking factors. Dogs were housed in triplets with others of the same diet group indoors with continuous access to a concrete outside pen. Between 9:00 and 14:30, the dogs also had access to a concrete outside paddock and they were socialized at least once a day (e.g. toy play or walk). During the last six days of each feeding period, dogs were housed individually with access to the outside paddock during the day. The average age, body weight and energy intake in kJ/kg BW^0.75^ per dog group are shown in Table 1.

**Table 1:** Gender, age, body weight and energy intake of the study dogs per dog group.

				Age (years)			Body Weight (kg)			Energy Intake (kJ/kg BW^0.75^)		
Group	n	♂	♀	Mean	Min	Max	Mean	Min	Max	Mean	Min	Max
A	6	3	3	4.7	2.8	8.1	28.6	26.3	32.7	440	360	561
B	6	2	4	3.7	2.3	5.9	28.3	26.6	32.2	443	360	645
C	6	1	5	5.2	3.4	6.7	27.3	23.7	31.5	414	360	578
D	6	3	3	3.3	2.0	5.0	27.3	23.7	31.0	443	360	722

Kg, kilogram; kJ, kilojoule; BW^0.75^, metabolic body weight; n, number of animals; ♂, male; ♀, female; Min, minimum; Max, maximum

### Diets

Eight commercially available diets were selected that varied in diet type (canned or kibble) and protein concentration (Table 2). Commercial diets were chosen for practical relevance of the study, and the difference in Se concentration and other parameters (e.g. ingredients, processing conditions, Se species) between the canned and kibble diets are considered inherent to the diet types. For every diet type, diets with an estimated (from the pet food labels) crude protein concentration of 9.6, 14.3, 19.1 and 23.9 g CP/MJ ME (40, 60, 80 and 100 g CP/1000 kcal ME, resp.) were included. Four different protein concentrations were selected, rather than diets with different protein digestibility coefficients. This resulted in a range of the absolute amount of digestible protein, as the limited differences in protein digestibility in commercially available diets are usually overruled by the protein concentration *per se*. All diets were single-batch, to prevent differences in nutrients over time due to variations in ingredients. Selected canned diets were Royal Canin^®^ Canine Veterinary Diet Hepatic, Sensitivity Control Duck & Rice, and Recovery and Canine Veterinary Care Nutrition Senior Consult Mature. Selected kibble diets were Royal Canin^®^ Canine Veterinary Diet Renal, Gastro Intestinal Moderate Calorie, and Satiety Weight Management and Canine Veterinary Care Nutrition Pediatric Junior Large Dog. The canned diets contained Se solely from the ingredients, which is primarily organically-bound Se [34], whereas Se in the form of sodium selenite was supplemented in all kibble diets before processing (standard, not specifically for this study). Supplemented amounts of sodium selenite were 0.05, 0.08, 0.08, and 0.08 mg/kg, respectively. As veterinary diets were used in this study, some of the diets were supplemented with additional nutrients in order to be nutritionally complete and balanced for healthy adult dogs with energy requirements of 397 kJ (95 kcal)/kg BW^0.75^. Details of the supplementation are included in Table 2. Each dog was fed individually to maintenance requirements.

**Table 2:** Analysed chemical composition (g/MJ ME, except where specified), dry matter (DM) and metabolisable energy concentration (ME) of four canned and four kibble single batch diets^a^ with differing protein concentrations.

	Canned				Kibble			
Calculated protein concentration*	9.6^b^^,^^d^^,^^h^	14.3^c^^,^^f^	19.1^b^^,^^d^^,^^f^	23.9^c^^,^^e^^,^^g^	9.6^c^^,^^i^	14.3	19.1	23.9
DM (g/100g as is)	36.1	30.2	24.9	26.9	92.8	91.0	91.4	91.0
Crude protein	10.1	15.0	21.5	27.5	11.3	14.3	18.5	23.2
Crude fat	9.2	11.0	11.3	13.0	9.2	7.6	10.1	8.1
Total dietary fibre^†^	4.7	4.3	4.5	6.1	4.5	4.6	4.0	26.6
Crude ash	2.8	5.5	3.4	3.7	2.8	4.3	4.7	4.7
Selenium (μg/MJ ME)	34.8	39.3	47.5	40.7	13.9	25.5	19.4	30.3
Iodine (μg/MJ ME)	255.7	138.6	74.1	14.3	219.6	374.4	300.4	348.9
d-α-tocopherol (mg/MJ ME)	24.0	21.6	20.7	21.1	29.0	31.6	32.4	83.5
ME (MJ/kg DM)^‡^	17.5	17.5	17.7	18.2	17.9	16.8	17.5	11.6
*Amino acids*								
Arginine	0.52	0.86	1.04	1.61	0.73	0.92	1.05	1.07
Histidine	0.22	0.34	0.59	0.51	0.24	0.29	0.33	0.44
Isoleucine	0.36	0.58	0.70	0.86	0.41	0.55	0.65	0.81
Leucine	0.81	1.09	1.88	1.63	1.07	1.05	1.35	1.71
Lysine	0.41	0.94	1.18	1.51	0.49	0.71	0.83	0.92
Methionine	0.24	0.41	0.38	0.57	0.23	0.29	0.49	0.41
Cysteine	0.13	0.11	0.18	0.18	0.18	0.20	0.27	0.36
Phenylalanine	0.43	0.60	1.04	0.92	0.53	0.59	0.74	1.00
Tyrosine	0.36	0.47	0.68	0.65	0.40	0.44	0.48	1.18
Threonine	0.36	0.58	0.90	0.88	0.37	0.53	0.61	0.74
Tryptophan	0.11	0.19	0.23	0.20	0.12	0.14	0.14	0.20
Valine	0.49	0.69	1.18	1.12	0.49	0.73	0.84	0.98
*Fatty acids*								
Linoleic acid	2.78	1.23	1.53	1.95	1.84	1.26	1.55	1.61
Arachidonic acid	0.04	0.20	0.18	0.14	0.04	0.04	0.04	0.05
Alpha-linolenic acid	0.09	0.10	0.13	0.15	0.16	0.10	0.13	0.14
Docosahexaenoic acid	0.01	0.17	0.20	0.32	0.05	0.03	0.03	0.07
Eicosapentaenoic acid	0.00	0.28	0.31	0.49	0.08	0.06	0.07	0.17

MJ, megajoule; ME, metabolisable energy

^a^ All diets were supplemented with 516 mg choline/dog/day, Choline chloride 78% solution, Taminco BVBA, Gent, Belgium

^b^ Supplemented with 89.7 mg magnesium/dog/day, Super magnesium, Metabolics Ltd, Eastcott, Wiltshire, England

^c^ Supplemented with 206.3 mg magnesium/dog/day, Super magnesium, Metabolics Ltd, Eastcott, Wiltshire, England

^d^ Supplemented with 2.2 mg copper/dog/day, Copper citrate, Metabolics Ltd, Eastcott, Wiltshire, England

^e^ Supplemented with 4.4 mg copper/dog/day, Copper citrate, Metabolics Ltd, Eastcott, Wiltshire, England

^f^ Supplemented with 3.8 μg vitamin D/dog/day, Pet-Cal^™^, Pfizer Animal Health, New York, USA

^g^ Supplemented with 0.43 mg iodine/dog/day, Iodine 11, Metabolics Ltd, Eastcott, Wiltshire, England

^h^ Supplemented with 1.19 g methionine/dog/day, synthetic methionine, Evonik Industries, Essen, Germany

^i^ Supplemented with 1.03 g methionine/dog/day, Evonik Industries, Essen, Germany

* The diets are from left to right: Royal Canin^®^ Canine Veterinary Diet Hepatic, Sensitivity Control Duck & Rice, Canine Veterinary Care Nutrition Senior Consult Mature, Canine Veterinary Diet Recovery, Renal, Gastro Intestinal moderate calorie, Canine Veterinary Care Nutrition Pediatric Junior Large Dog and Canine Veterinary Diet Satiety Weight Management

^†^ Obtained from the pet food producer

^‡^ Calculated using predictive equations for ME [21]

### Blood samples

At the end of every feeding period, blood samples (13ml) were taken at 13:00 h from each dog by jugular venipuncture using a 21 gauge needle. Blood was put into 2 Microvette^®^ 500 μl lithium-heparin tubes, one 300 μl fluoride-heparin tube, one 200 μl Tri-Kalium-EDTA tube and 2 Vacuette^®^ z serum clot activator tubes (1× 9 ml and 1× 4 ml). One of the lithium-heparin tubes was used for whole blood GPx analysis immediately after collection. The remaining heparin tubes were centrifuged (accuSpin^™^ Micro R, Thermo Fisher Scientific Inc.) immediately after collection at 1680 g and 4°C for 10 min. Lithium-heparin plasma was analysed for general biochemistry parameters and fluoride-heparin plasma for glucose (Spectrophotometry, Olympus AU400). Tri-Kalium-EDTA tubes were placed on a roller at room temperature until analysis for haematology parameters (Mythic 18 Vet analyser, Orphée S.A.). Biochemistry and haematology measurements were used as a general health check to confirm that all dogs were in good health. Serum tubes were incubated for 30 min on ice and then centrifuged (Sigma 6K15, rotor 11150, cups 13550, Sigma GmbH) for 10 min. at 2000 ×g and 4°C. Serum samples were divided into centrifuge tubes and stored at -80°C for analysis of Se, IsoPs and triiodothyronine (T3) and thyroxine (T4) at the end of the study.

### Urine samples

Free catch urine was collected in the section of the day between meals at the end of each feeding period, using a Uripet urine collection device (Rocket Medical plc., Watford, England). 1 ml of urine was stored in at -80°C and analysed for creatinine (CT) within one month after sampling (IDEXX laboratories, UK). The rest of the sample was stored at -20°C and analysed for Se content (ICP-MS).

### Faeces samples

During the last six days of each feeding period, titanium dioxide (TiO_2_) was added to the diets as a digestibility marker at a concentration of 0.45 g/day (mean 1.5 g/kg DM, range 0.67–2.14 g/kg DM). Total faeces were collected during four consecutive days at the end of each diet phase. Faeces were homogenised with a mixer (Kenwood professional PM900, Kenwood LTD) for 1 minute and a sample of approximately 350 g (mean 346, range 245–410 g) was taken. Samples were freeze-dried (SuperModulyo^®^, Thermo Fisher Scientific Inc.) until a stable weight, at -50°C. Faeces were analysed for TiO_2_, DM, ash, nitrogen (N) and Se. N and Se were used for the calculation of apparent Se and CP (N×6.25) digestibility coefficients, respectively, with the use of TiO_2_ as a marker by means of the following formula:

$$\text{Apparent digestibility \%}=100-(100\;\times \left(\frac{\text{\% marker in diet}}{\text{\% marker in faeces}}\;\times \;\frac{\text{\% nutrient in faeces}}{\text{\% nutrient in diet}}\right))$$

### Chemical analyses

Biochemistry and glucose analyses were carried out using spectrophotometry (Olympus AU400, Olympus Inc.) with Beckman Coulter reagents (Beckman Coulter Biomedical LTD) within 20 mins of sampling. Whole blood GPx was also analysed within 20 mins of sampling using the Ransel kit (Randox laboratories LTD) on the Olympus AU400 spectrophotometer. A 4-point calibration curve was used as control. Whole blood GPx analysis had an average CV of 1.4% in the samples and the 4-point calibration curve showed a recovery of 83.1, 93.7, 98.5, and 96.4%, respectively. Haematology parameters were analysed using a Mythic 18 Vet analyser (Orphée S.A.). Thyroid hormone analyses (T3 and T4) were performed according to the method of Darras et al. [35] and the IsoPs were analysed using an enzyme-linked immunosorbent assay (8-isoprostane EIA kit, Cayman Chemical Co.). Serum, urine and faeces samples were prepared for total Se analyses with closed vessel microwave destruction as described in van Zelst *et al*. [19]. Se was analysed using inductively coupled plasma-MS (ICP-MS, Elan DRC-e, PerkinElmer), as described by Lavu *et al*. [36]. Urine CT was determined using a creatinine kit based on the Jaffe reaction (OSR6178, Beckman Coulter Biomedical LTD, IDEXX Laboratories, UK). Faeces samples were analysed for DM and crude ash by drying to a constant weight at 103°C and combusting at 550°C, respectively. The Kjeldahl method (ISO 5983–1, 2005) [37] was used to determine CP (N×6.25). TiO_2_ was analysed according to the method of Myers *et al*. [38].

### Statistical analyses

Data were analysed using linear mixed effect models in RStudio (version 0.98.507, RStudio,Inc.) [39] using the nlme package [40]. Two primary response variables were measured: GPx and urinary Se:CT ratio relative to the Se intake (relative urinary Se:CT ratio). To account for two primary response variables, the statistical test level was corrected to 0.05/2 = 0.025 for these two parameters. The secondary response variables in this study were: Energy intake, Se intake, serum Se, serum IsoP, serum T3:T4 ratio, Se digestibility, apparent CP digestibility, digestible Se intake (the absolute amount of digestible Se), digestible CP intake and urinary Se:CT ratio relative to the digestible Se intake. For these response variables a *p*-value of <0.05 was used as the threshold for significance.

The model contained a fixed effects structure of actual CP intake (g/kg BW^0.75^), diet type and their interaction and a random structure of dog. If the interaction was not significant (*p*≥0.05), the model was reduced to CP intake and format as fixed effects and dog as random effect. Energy, Se, digestible Se and digestible CP intake were confounded with CP intake, and therefore CP intake was omitted from the model for these parameters. Distributional assumptions were checked by visual inspection of residuals. Results are reported as means (± SEM).

To assess the impact of time between a dog’s last meal and urine collection on the relative urinary Se:CT ratio, a likelihood ratio test was carried out by including time into the fixed effects structure of the model. No significant differences were found when time was included as a parameter; consequently, time was omitted from the structure of the mixed effects model for relative urinary Se:CT ratio.

## Results

All biochemistry and haematology results were within the normal range for healthy dogs (data not shown). The average Se concentration of the kibble diets was 22.3 μg/MJ (range 13.9–30.3) and 40.6 μg/MJ (range 34.8–47.5) for canned diets. Energy intake did not differ between the diet types, but Se intake was lower on kibble than canned diets (Table 3).

**Table 3:** Energy, selenium and crude protein intakes per diet type of four canned and four kibble diets.

	Canned		Kibble		*p*-value
	mean	SEM	mean	SEM	diet type
Energy intake (kJ/ kg BW^0.75^)	428.8	11.7	442.9	11.7	0.325
Se intake (μg/kg BW^0.75^)	17.3	0.4	10.1	0.6	<0.001
Digestible Se intake (μg/kg BW^0.75^)	7.7	0.4	6.6	0.4	0.043
Digestible CP intake (g/kg BW^0.75^)	6.6	0.5	6.5	0.3	0.860

SEM, standard error of the mean; BW^0.75^, metabolic body weight; Se, selenium; CP, crude protein.

**Fig 1:**
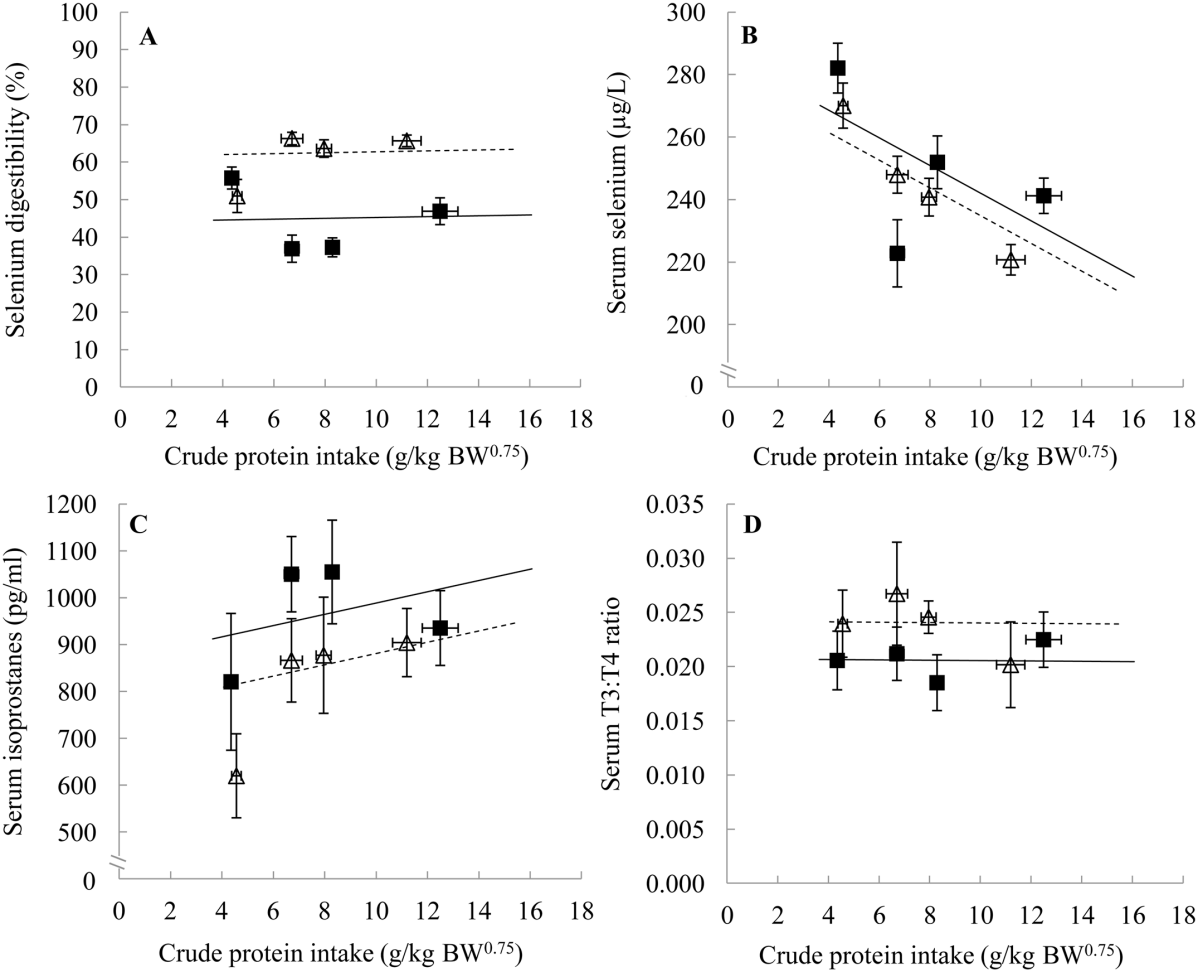
Selenium digestibility (A), serum selenium (B), serum isoprostanes (C) and serum T3:T4 ratio (D) in dogs in relation to crude protein intake of four canned and four kibble diets. Black squares and solid line are canned diets, open triangles and dashed line are kibble diets. Symbols represent the means and error bars indicate their standard errors, based on the raw data. Lines are based on the linear mixed model estimates. BW^0.75^, metabolic body weight; T3:T4 ratio, ratio between triiodothyronine and thyroxine hormones.

Apparent CP digestibility showed a significant diet type by CP intake interaction (*p*<0.001). Apparent CP digestibility increased with increasing CP intake, but only in canned diets. The digestible CP intake did not differ between diet types (Table 3). Kibble diets had a higher Se digestibility on average than canned diets (*p*<0.001) and Se digestibility did not significantly change with CP intake (*p* = 0.753, Fig 1A). On average, Se digestibility was 62.3% (± 1.5) in kibble and 44.9% (± 1.9) in canned diets. The average digestible Se intake was lower in kibble than canned diets (Table 3).

**Fig 2:**
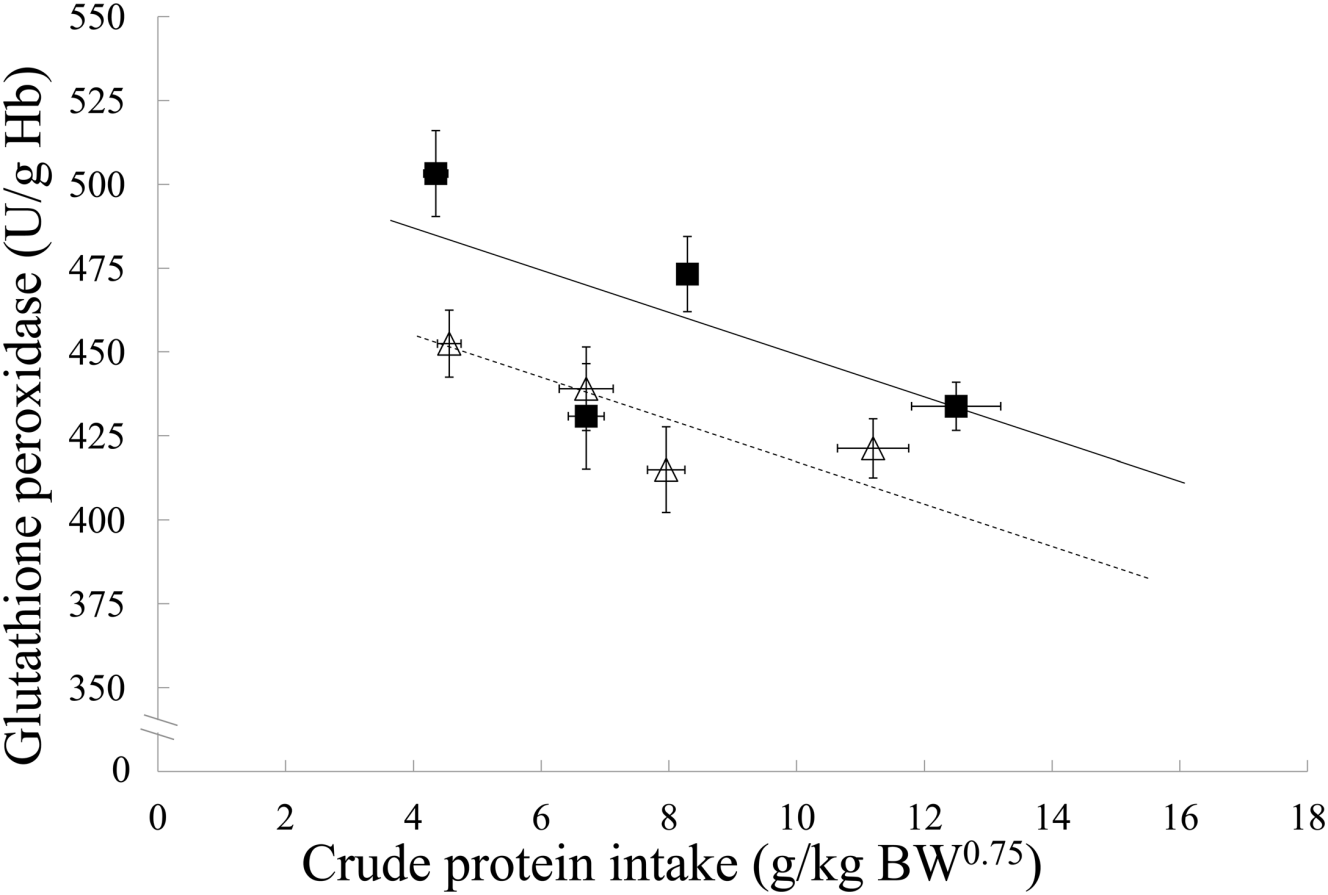
Whole blood glutathione peroxidase responses in dogs in relation to crude protein intake of four canned and four kibble diets. Black squares and solid line are canned diets, open triangles and dashed line are kibble diets. Symbols represent the means and error bars indicate their standard errors, based on the raw data. Lines are based on the linear mixed model estimates. Hb, hemoglobin; BW^0.75^, metabolic body weight.

The GPx response was lower on average in kibble compared to canned diets (*p*<0.001) and decreased with an increasing CP intake (*p*<0.001, Fig 2). Serum Se concentrations did not change significantly with diet type (*p* = 0.158), but did with CP intake, whereby serum Se concentrations decreased with increasing CP intake (*p*<0.001, Fig 1B). No association of diet type (*p* = 0.069) or CP intake (*p* = 0.216) on IsoPs was found (Fig 1C), nor with the T3:T4 ratio (*p* = 0.102 and 0.960, respectively, Fig 1D).

**Fig 3:**
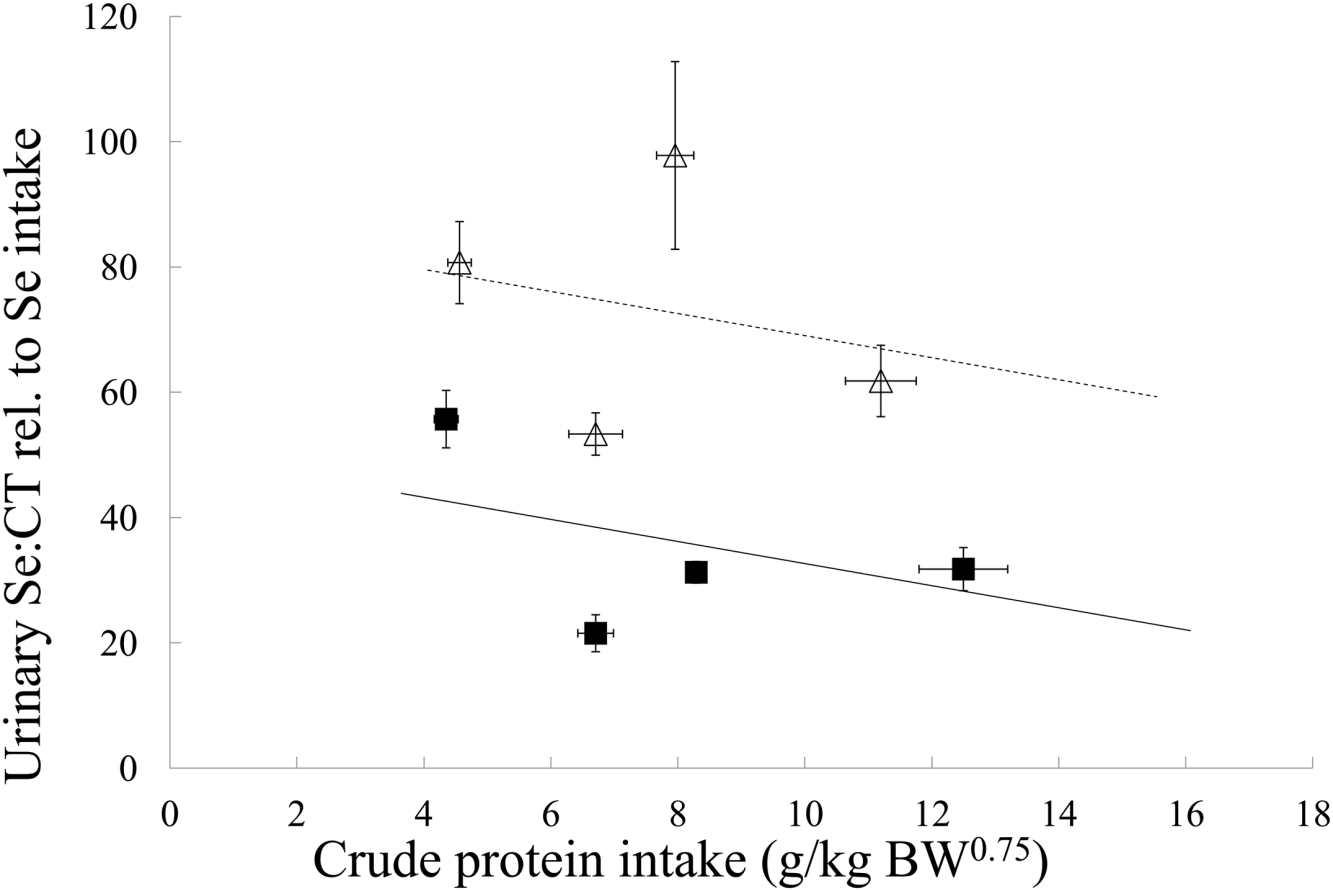
Urinary selenium to creatinine ratio relative to selenium intake in dogs in relation to crude protein intake of four canned and four kibble diets. Black squares and solid line are canned diets, open triangles and dashed line are kibble diets. Symbols represent the means and error bars indicate their standard errors, based on the raw data. Lines are based on the linear mixed model estimates. BW^0.75^, metabolic body weight.

The relative urinary Se:CT ratio was higher on average in kibble diets than in the canned diets (*p*<0.001). No association of CP intake with urinary Se:CT ratio was found (*p* = 0.059, Fig 3). When urinary Se excretion was expressed per absolute amount of digestible Se, dogs on kibble diets excreted more Se in their urine than dogs on canned diets (*p* = 0.001) and excretion decreased with increasing CP intake (*p* = 0.017, data not shown).

## Discussion

This study showed that the availability of Se through digestion and metabolism changed considerably with diet type. The higher apparent Se digestibility in kibble compared to canned diets suggests that the use of a single recommendation for Se inclusion levels in pet foods for both diet types needs reconsideration. Previous *in vitro* findings showed that Se in canned diets was more susceptible to a decrease in Se accessibility, i.e. the amount that is available for absorption in the gastro-intestinal tract, due to processing [19]. The average Se accessibility was also lower in canned than kibble diets [19]. In the *in vitro* study [19], canned diets had an average Se accessibility of 58% (± 4.01, n = 20), while the Se accessibility of kibble diets was 72% (± 3.95, n = 23). Accordingly, in the present study canned diets showed a lower Se digestibility than kibble diets. The difference in apparent Se digestibility observed between the diet types may be caused by the Se species used in these diets. The meat and meat-by-products that are used in canned diets, mainly contain organically bound Se (e.g. selenomethionine) [41]. Therefore, it may be that the dietary sulphur-containing amino acids (methionine and cysteine) compete for absorption with the organically bound Se [42]. Sodium selenite, which is often used (as in the kibble study diets) to supplement pet foods that do not comply to the recommended allowance (mainly kibble diets), is absorbed through diffusion [43] and thus does not have to compete for absorption with organically bound sulphur.

Interestingly, bioactivity in this study measured as GPx, was higher in canned than kibble diets. This may be explained by a higher amount of digestible Se in canned diets. Even though the apparent Se digestibility of canned diets was much lower than kibble diets (44.9% vs. 62.3%, respectively), the absolute amount of digestible Se was still higher in canned diets (7.7 ± 0.4 vs. 6.6 ± 0.4 μg/kg BW^0.75^, respectively). Both this study and the *in vitro* study [19] were performed with commercially available diets and in both studies a higher dietary Se concentration was found in canned than kibble diets. In the *in vitro* work, canned diets contained on average 34.8 μg Se/MJ (± 6.25, n = 20) and kibble diets 22.5 μg Se/MJ (± 2.42, n = 23) [19]. In this study, similar contents were measured (canned diets: 40.6 μg Se/MJ, kibble diets: 22.3 μg/MJ). This indicates that the ingredients for canned diets tend to contain a higher amount of Se, which suggests this may be inherent to this diet type.

In addition to the higher amount of digestible Se in canned diets, the urinary Se excretion relative to Se intake was lower in canned compared to kibble diets, even per absolute amount of digestible Se. This suggests that the percentage of retained Se per digestible Se is higher in canned than kibble diets. Consequently, a higher amount of digestible Se from the canned diets can be used for either non-specific incorporation into body proteins, or incorporation into the Se-dependent antioxidant GPx. In the latter situation, this results in a higher bioactivity of Se from canned diets, as demonstrated in this study. A difference in speciation is the most likely explanation for the differences between the diet types. Unfortunately, efforts to determine Se speciation were unsuccessful due to detection limit issues. However, it is known that the kibble diets in this study are supplemented with inorganic sodium selenite and that the Se in the canned diets in this study derives solely from the ingredients, which consist mainly of meat and meat by-products, and thus it is primarily organically bound [41].

It should be acknowledged that apparent digestibility was calculated in this study. Some of the absorbed Se is excreted via the bile into the faeces [44], which may cause an underestimation of the Se digestibility. However, it is not known whether there was a difference in biliary Se excretion between the diet types. Given the higher dietary fat concentration in canned diets, it may be assumed that bile excretion is higher in canned compared to kibble diets, but this does not necessarily mean a higher Se excretion via bile. Gregus *et al*. [45] suggest that Se excretion via bile is enhanced by binding to an organic acid. So, if there was a difference in the amount of Se excreted via the bile, this may also be due to a difference in Se speciation.

Taking blood GPx concentration as a measure for bioactivity may suggest a superior antioxidant function of canned diets, but this was not associated with a higher concentration of the oxidative stress parameter, isoprostanes. It cannot be excluded that a difference between diet types may be found if other parameters of oxidative stress were measured or if IsoP’s were measured during a longer period.

Like GPx, iodothyronine deiodinases are also Se-dependent hormones. Several studies have shown that a higher dietary Se concentration has a positive influence on the T3:T4 ratio in humans [32], kittens [16, 31] and dogs [16, 46]. In this study, no effect on T3:T4 ratio was found and they were shown to be within a clinically normal range [47]. This may be because the difference in digestible Se was not large enough between diet types. Remarkably, no interactions were found for any of the parameters measured in this study. However, there was a negative association between CP intake and both GPx and serum Se, irrespective of diet type. It is unlikely that Se intake was responsible for these results as there was no negative, but actually a positive association between Se and CP intake. Se digestibility was also not significantly associated with CP intake, therefore, unlikely to be responsible for the negative association between bioactivity and CP intake. Furthermore, urinary Se excretion corrected for digestible Se decreased with increasing CP intake, which suggests a higher retention with increasing CP intake. The higher amount of retained Se was not incorporated into GPx, but left in the blood stream as serum Se or excreted via the urine. Therefore, taken together it is very likely that a part of the Se from canned diets was incorporated into body proteins as selenomethionine instead of methionine.

To clarify whether the effects found in this study were attributed to the absolute amount of Se or the Se species, semi-purified diets could be used in the future. However, it is a challenge and maybe even impossible to manufacture diets with two different processing types, using exactly the same ingredients. Furthermore, such a study would not be relevant for the Se digestibility and bioactivity of commercially available diets. This implies that the effects of CP intake in this study include factors associated with the formulation of a diet to a certain CP concentration. Similarly, the effect of diet type is not just a result of processing, but includes the choice of ingredients for that particular diet type.

In conclusion, the diet type, whether this is caused by processing, Se speciation, or otherwise, causes canned diets to have a lower apparent Se digestibility, but a higher amount of the digestible Se is retained in the body and thereby enhancing Se bioactivity. So the can can, what the kibble can't. This study suggests that the recommendation for Se inclusion levels in pet foods should take diet type into account. Further studies are warranted to quantify the exact origin of the difference between the diet types, but this requires valid and sensitive parameters to monitor Se adequacy. The impact of processing conditions and protein sources on Se digestibility and bioactivity is another topic that warrants more investigation. It should be noted that it is not sufficient to solely measure Se digestibility, because bioactivity and urinary excretion measurements may give a different picture. Therefore, the choice of parameters can be crucial for the conclusion when studying nutritional modulation of Se status in animals.
